# Supplementary material for: Are carbon emissions trading and green financial instruments synergistic? -Comprehensive quantitative research based on content analysis
Source: PLoS One. 2024 Mar 7;19(3):e0298601. doi: 10.1371/journal.pone.0298601 (PMC10919601; doi:10.1371/journal.pone.0298601)
Supplement: S1 Data — (ZIP) [file pone.0298601.s001.zip › Data/地级市Z_F工作报告.zip!/╔╜╢1⁄2/╫═▓⌐╩╨/└┤╫╘╣1⁄2╓┌║┼í╛┬φ┐╦╩2╛▌═°í┐_2008.docx]

各位代表：

　　现在，我代表市人民政府，向大会作政府工作报告，请予审议，并请各位政协委员和其他列席会议的同志提出意见。

务实奋进、跨越发展的五年

　　过去五年，我市各级政府和全市人民在中共淄博市委的坚强领导下，坚持以邓小平理论和“三个代表”重要思想为指导，以科学发展观统领全局，在历届市领导班子打下的良好基础上，努力推动科学发展、和谐发展、率先发展，全市经济社会实现新的跨越，　　“十五”计划顺利完成，市十二届人大一次会议确定的各项目标全面实现，建设殷实小康、构建和谐淄博实现良好开局。

　　一、综合经济实力大幅跃升。2007年，全市地区生产总值预计达到1920亿元以上，是2002年的2.6倍，年均增长16%以上，人均生产总值达到43670元，提前两年实现比2000年翻一番的目标。境内财政收入完成245.5亿元，是2002年的2.96倍，年均增长24.2%。地方财政收入完成97.78亿元，是2002年的2.95倍，年均增长24.2%。税收和地方财政收入占GDP的比重分别为11.55%和5.09%，比2002年分别提高1.3个和0.59个百分点。累计完成全社会固定资产投资2468亿元，是上一个五年的3.1倍。预计全市金融机构存款余额达到1388亿元，是2002年的2.06倍，年均增长15.55%；全社会消费品零售总额达到588亿元，是2002年的2.1倍，年均增长16%。经济增长质量和效益不断提高。

　　二、老工业城市转型成效显著。工业结构优化升级步伐明显加快，总量规模和整体竞争力显著增强。预计2007年，全市规模以上工业销售收入、利税、利润分别达到4420亿元、482亿元、289亿元，分别是2002年的4.3倍、5.4倍和7.9倍。全市销售收入过百亿、过50亿、过30亿的工业企业分别达到4户、9户和12户。加快推进创新型城市建设，成功创建国家新材料成果转化及产业化基地、国家生物医药产业基地、国家泵类产业基地和国家先进陶瓷产业基地。被国家确定为知识产权试点城市。省级以上工程技术研究中心和企业技术中心达到112家，其中国家级5家。承担了一大批国家和省级科技计划项目，东岳集团的离子膜项目被列入“十一五”国家科技支撑计划重大项目。全市高新技术产业产值达到1410亿元，占规模以上工业总产值的比重达到32.1%，比2002年提高10.8个百分点。非公有制经济快速发展，占国民经济比重达到61%。中国名牌、中国驰名商标总数分别达到30个、17件，国家免检产品85件，均居全省前列。获准筹建国家陶瓷与耐火材料产品质量监督检验中心。12个经济园区通过国家审核，产业集聚发展和布局优化得到加强。现代服务业蓬勃发展，一批国内外著名连锁企业落户淄博，预计2007年服务业完成增加值607亿元，是2002年的2.3倍，占地区生产总值的比重达到31.6%。农业综合生产能力稳步提高，产业化、标准化、基地化建设加快推进，畜牧、林果、蔬菜三大主导产业地位更加巩固。

　　三、节能减排和环境保护切实加强。启动实施生态市建设规划和碧水蓝天行动计划，完成环保投资146.8亿元，是前五年的2.62倍，建成城市污水处理厂12座，城市污水集中处理率达到85%。加大对重点流域、区域和重点部位环境综合整治力度。严肃环保执法，严格“一票否决”，对环境违法行为实施“顶格处罚”。全市主要水体质量和大气质量显著改善。深入开展土地市场和矿业秩序整顿，国土资源管理进一步加强。加大生态建设力度，大力发展循环经济，狠抓节能降耗，关停了一批能耗高、污染重的“土小”企业，稳妥有序地淘汰落后产能，资源节约型、环境友好型社会建设迈出新步伐。2007年，全市化学需氧量、二氧化硫排放量削减任务有望完成，节能降耗工作成效明显。2007年以网上用电7.6%的增幅支撑了GDP15%的增长。

　　四、城乡面貌发生巨大变化。按照“中心凸显、十字展开、组团发展”的思路，做大做强做美做畅中心城区，有序启动新城区建设，协调推进组团城区与小城镇建设，初步构筑起现代化城市发展的总体框架。建成区面积由2002年的154平方公里扩大到200平方公里，城市化率由53%提高到61%，城市绿化水平明显提高。完成了昌国路立交桥、张博路附线、济青高速南线、胶济铁路电气化改造、市垃圾焚烧发电厂等一大批基础设施，城市功能显著增强，城市规划、建设、管理水平显著提高。务实推进社会主义新农村建设，全面实施建设农村殷实小康十大工程，加大“三农”投入，村村通油路、通客车、通自来水、通广播电视等目标基本实现，农村教育、卫生条件得到显著改善。

　　五、改革开放向纵深推进。深化企业改革，全市大中型企业普遍建立起现代企业制度。全面完成上市公司股权分置改革，五年全市新增上市企业4家，期间实现直接融资73.5亿元。对400余家企业实施了破产、重组。以行政审批制度改革、相对集中行政处罚权为主要内容的行政执法体制改革实现新突破。公共财政体系建设以及部门预算、国库集中支付、政府采购等改革迈出新步伐。粮食流通体制改革全面完成。农村综合改革逐步深化，农业税、农业特产税、“三提五统”全部取消，涉农补贴等支农惠农政策全面落实。金融、投资、流通等各项改革稳步推进。市商业银行获准在市外设立分支机构。对外开放水平进一步提高，五年共引进境内外资金725亿元，其中，利用外商直接投资17.3亿美元、年均增长18.1%，9家世界500强企业落户淄博。预计完成进出口总额160.6亿美元，其中出口99.6亿美元，年均增长31.7%。对外经济技术交流与合作不断扩大，企业“走出去”迈出新步伐。中国(淄博)国际陶瓷博览会和新材料技术论坛等会展的品牌影响力逐步扩大。

　　六、社会事业繁荣进步。大力实施创建文明城市“四大工程”，深入开展群众性精神文明创建活动，加强社会主义荣辱观教育，市民素质和城市文明程度进一步提高。坚持教育的普惠性和公益性，基础教育基本完成十五年教育普及任务，高中段教育普及率达到95%，高等院校增加到12所。成功战胜“非典”、“禽流感”等重大疫情，基本建立起突发公共卫生事件预防控制和农产品、食品、药品质量安全监管体系。文化事业繁荣进步，文化产业加快发展。抑制第四次人口出生高峰，稳定了低生育水平。全民健身和竞技体育水平进一步提高，实现了奥运金牌零的突破，被国际足联确认为世界足球运动起源地，成功申办22届省运会。积极推进依法治市，正确处理新形势下的人民内部矛盾，深化社会治安综合治理，严厉打击刑事犯罪和“法轮功”等邪教组织，平安淄博建设成效显著。防灾减灾体系逐步完善，应急管理体制初步建立。一刻也不放松地抓好安全生产，保持了安全生产形势的稳定。审计、监察、统计、物价、广播电视、新闻出版、民族宗教、外事、侨务、对台、人防、气象、防震、史志、档案、仲裁、老龄、残疾人、红十字、民兵预备役等各项事业都有新进步。

　　七、民生得到显著改善。2007年预计城市居民人均可支配收入、农民人均纯收入、在岗职工平均工资分别达到15330元、6442元和22919元，分别是2002年的1.95倍、1.77倍和1.9倍;城乡居民人均储蓄存款达到1.9万元，是2002年的2.11倍;居民衣食住行用水平不断提高，消费结构进一步优化。五年累计建设经济适用房164.09万平方米。实施了农村残疾人安居工程。实施积极就业政策，不断完善社会保障体系。五年累计减免税费和落实再就业补贴3.36亿元，新增就业再就业54万人(次)，转移农村劳动力41.5万人(次)，城镇登记失业率由2002年的3.6%下降到2.6%。五大保险参保职工累计达到366万人(次)，社保基金累计结余50亿元，分别是2002年的2倍和7.5倍，累计支付各项社保资金124亿元。城乡低保标准逐步提高，实现应保尽保。农村五保集中供养率达到70%。新型农村合作医疗实现全覆盖，参合农民229.91万人，参合率达到91.37%。全面启动城镇居民基本医疗保险试点和做实企业养老保险个人账户工作。积极探索解决了3万多名国有破产企业退休职工参加基本医疗保险问题。城乡义务教育阶段免费教育全面实现。安排专项资金用于家庭困难学生补助。大中专院校学生助学金以及水库移民后期扶持、猪肉价格补贴等各项惠民政策全面落实。

　　各位代表！过去的五年，是淄博经济社会发展史上最好最快的时期之一。这五年，是经济实力显著提升、人民群众得到更多实惠的五年，是人与自然日趋和谐、城乡面貌显著变化的五年，是统筹协调全面加强、社会全面进步的五年。五年来，淄博先后荣获中国优秀旅游城市、中国陶瓷名城、国家园林城市、中国投资环境五十优城市、双拥模范城六连冠、省级文明城市、中国优秀创新型城市等荣誉称号。过去五年的成就，为今后发展打下了坚实基础，过去五年的实践，为继续前进积累了宝贵经验。五年来，我们始终坚持发展第一要务，牢固树立和落实科学发展观，认真贯彻执行国家宏观调控政策，着力把握发展规律、创新发展理念、转变发展方式、破解发展难题，（上接第一版）推动经济社会发展向又好又快转变，取得了科学发展的重大成果；始终坚持改革开放，解决了一批事关长远和全局的深层次矛盾和问题，引进了一批战略投资伙伴，为经济社会发展注入了新活力；始终坚持以人为本，正确处理改革发展稳定的关系，高度关注民生，着力解决人民群众最关心、最直接、最现实的利益问题，让人民在发展中得实惠，使社会在和谐中求进步;始终坚持以创新的精神加强政府自身建设，按照“为民、务实、清廉”的要求，扎实推进服务型政府建设，大力倡导“认真、专业、务实”政风，为发展提供了坚实保障。

　　各位代表！过去五年成绩来之不易。这是中共淄博市委正确领导的结果，是全市上下奋力拼搏的结果，是市人大、市政协和社会各界有效监督、大力支持的结果。在此，我代表市人民政府，向为淄博现代化建设做出贡献的全市人民，向各位人大代表、政协委员，向各民主党派、工商联、各人民团体和各界人士，向驻淄部队、武警官兵和中央、省驻淄单位，向关心支持淄博发展的海内外各界朋友，表示衷心的感谢和崇高的敬意！

　　在肯定成绩的同时，我们也清醒地看到，我市经济社会发展还面临一些困难和挑战，工作中还存在一些问题和不足。主要是：经济结构性矛盾和粗放发展方式尚未根本改变;资源环境和要素约束趋紧，节能减排压力巨大；制约发展的体制机制性障碍依然突出，市场化程度不高;发展的不平衡性依然存在，统筹发展任务艰巨；就业结构性矛盾突出、压力加大，部分群众生活还比较困难，和谐社会建设面临许多新情况、新问题；干部作风和机关效能还不尽如人意，政府自身建设有待进一步加强，等等。对存在的这些问题和不足，我们一定高度重视，采取更加有力的措施，切实加以解决。

　　今后五年工作的指导思想和奋斗目标

　　今后五年，是全面贯彻落实党的十七大精神，建设殷实小康、构建和谐淄博的关键时期。政府工作总的指导思想是：高举中国特色社会主义伟大旗帜，深入贯彻落实科学发展观，围绕建设殷实小康、构建和谐淄博奋斗目标，坚持发展第一要务，加快转变经济发展方式，深化改革扩大开放，在更高起点上实施科教兴市、结构优化、经济国际化、城市化和环境立市战略，更加突出结构调整、节能降耗和环境保护，更加注重统筹协调发展，着力改善民生，努力实现社会主义现代化建设的新跨越。

　　按照市十次党代会确定的目标和上述指导思想，衔接“十一五”规划，今后五年的主要奋斗目标建议为：到2012年，全市国内生产总值达到3235亿元，年均增长11%，人均71000元以上；地方财政收入达到202亿元，年均增长16%；城市化水平达到70%以上；城市居民人均可支配收入达到25000元以上，年均增长9%；农民人均纯收入达到11000元以上，年均增长10%。主要约束性目标建议为：万元生产总值综合能耗年均降低5%，化学需氧量和二氧化硫排放量年均分别减少3.7%和6.1%，人口自然增长率控制在5.5‰以内。

　　全面贯彻落实党的十七大精神，是实现今后五年奋斗目标的根本保证。必须把学习和贯彻党的十七大精神作为当前和今后一个时期的首要任务，深刻领会实现全面建设小康社会奋斗目标的新要求，深刻领会科学发展观的科学内涵、精神实质和根本要求，增强落实科学发展观的自觉性和坚定性，以科学发展观统领经济社会发展全局，按照“科学发展、和谐发展、率先发展”的要求，加快殷实小康、和谐淄博建设步伐。在工作指导上坚持和把握好以下几个方面：

　　——必须坚持又好又快发展不动摇。科学发展观第一要义是发展。必须牢牢把握经济建设这个中心，聚精会神搞建设、一心一意谋发展，不断解放和发展社会生产力。必须加快工作指导转变，坚持好字优先，好中求快，又好又快，始终坚持把结构调整作为经济社会发展的主线，把环境保护作为经济社会发展的命门，把节能降耗作为经济社会发展的关键，切实把科学发展落实到经济社会发展的各个环节和具体工作项目上，不断提升淄博综合竞争力，为殷实小康、和谐淄博建设打下坚实基础。

　　——必须加快转变发展方式。准确把握工业化、信息化、城镇化、市场化、国际化深入发展带来的机遇和挑战，坚持走中国特色新型工业化道路，促进经济增长由主要依靠投资、出口拉动向依靠消费、投资、出口协调拉动转变，由主要依靠第二产业带动向依靠第一、第二、第三产业协同带动转变，由主要依靠增加物质资源消耗向主要依靠科技进步、劳动者素质提高、管理创新转变。大力发展高新技术产业，改造提升传统产业，加快发展现代服务业和现代农业，构筑现代产业体系。

　　——必须坚持统筹协调发展。牢牢把握统筹兼顾这一根本方法，从区域发展不平衡的实际和组群城市的特点出发，更加注重城乡统筹和区县域经济发展，坚持以工促农，以城带乡，城乡互动；正确处理城市建设与新农村建设、中心城区与组团城区建设、城乡建设与产业发展的关系，推动经济社会全面协调可持续发展。

　　——必须最大限度启动改革开放两大动力。坚定不移地把改革创新作为推动经济社会发展的根本动力，提高改革措施的科学性，增强改革措施的协调性，建立健全贯彻落实科学发展观的体制机制；在更高层次上发挥扩大开放对经济社会发展的推动作用，拓展对外开放的深度和广度，充分利用两种资源、两个市场，拓展发展空间，增强发展活力。

　　——必须坚持以人为本、民生优先。把实现好、维护好、发展好最广大人民的根本利益作为一切工作的出发点和落脚点，把充分就业、增加收入、完善保障作为政府工作的优先目标，维护社会公平正义，促进社会和谐，促进人的全面发展，做到发展为了人民、发展依靠人民、发展成果由人民共享。

　　促进国民经济又好又快发展

　　建设殷实小康、构建和谐淄博，关键要转变发展方式，破解发展难题，加快发展先进生产力。要积极抢抓机遇，切实加强主体功能区规划研究，准确把握在全省“一体两翼”发展布局中的定位，努力拓展科学发展的新空间。

　　一、扎实推进社会主义新农村建设。按照“城乡一体、统筹发展”的思路，以促进农民增收为核心，深入实施建设农村殷实小康十大工程，建立以工促农、以城带乡长效机制，努力形成城乡经济社会发展一体化新格局。大力发展现代农业。坚持标准化、基地化、产业化发展方向，稳定发展粮食生产，培育壮大畜牧、林果、蔬菜三大主导产业，大力发展无公害、绿色、有机农产品，打造有机农产品基地品牌。深入推进农业产业化，大力发展农村合作经济组织，培植壮大100家重点龙头企业，提升农业竞争力。健全农村市场和农业服务体系，推动农业科技进步，提高农业机械化水平。加强农业综合开发，抓好国家农业综合开发示范区建设。加强动植物疫病防控，提升农产品质量安全水平，全市主要农产品全部达到无公害、绿色或有机标准。加强农村基础设施建设，全面实施大中小型水库除险加固，加快推进大中型骨干河道综合治理。完善提高“村村通”工程。积极推进小城镇建设。坚持城乡发展规划“一张图”，科学调整镇村布局，突出发展中心镇、城镇组团和中心村，积极稳妥地推进“合村并居”，促进工业向园区集中、人口向城镇集中、居住向社区集中，形成一批富有实力的中心镇和特色鲜明的农村社区。突出解决农村垃圾处理、道路硬化等实际问题，推进生态家园富民工程，着力改善农村人居环境。加快发展农村二、三产业，实现城镇化与工业化良性互动。全面落实农村各项政策。巩固、完善各项支农惠农政策，建立支农投入稳定增长机制，促进公共资源向“三农”倾斜，实现城乡基本公共服务均等化。稳定和完善土地承包经营制度，按照依法自愿有偿的原则，促进土地承包经营权合理有序流转，发展多种形式的适度规模经营。深化农村综合改革、土地征用制度改革、林权制度改革。积极推进农村金融产品和服务创新，扩大农业政策保险实施范围。继续做好扶贫开发、库区移民等工作。

　　二、加快构筑现代产业体系。按照全省“一体两翼”发展布局，坚持走新型工业化道路，努力形成以高新技术产业为先导、先进制造业为支撑、服务业全面发展的产业格局，努力打造现代化工业名城和区域性现代服务业中心城市。推进工业结构优化升级。要遵循国家产业政策导向，充分发挥比较优势，建设高水平的国家新材料产业化基地、国家生物医药产业化基地和全国重要的石油化工基地。经过五年努力，使石油化工、机电装备、新材料产业年销售收入分别达到1500亿元、1100亿元、1000亿元规模，精细化工、信息、医药产业分别达到800亿元、500亿元、300亿元规模。积极运用先进技术，对机电、纺织、冶金、建材、轻工等传统产业普遍进行改造，力争完成技改投入1500亿元以上，使规模以上企业70%以上的技术装备达到国际国内先进水平，能耗和污染排放全部达到国家标准。整合要素资源，集中支持发展一批拥有自主知识产权、核心竞争力强的大企业、大集团，组织实施一批市场前景好、科技含量高、符合国家产业政策的重点项目。完善中小企业服务体系，鼓励中小企业与大企业开展协作配套。实施质量兴市和名牌带动战略，培育一批知名品牌。优化产业布局，充分发挥12个重点经济园区的产业聚集作用，逐步形成具有特色优势的产业集群。制定重点发展、限制发展和加快转移的产业、产品、技术和生产装备目录，限制和淘汰落后产能。全面完成电力工业“上大压小”任务，新上先进环保发电机组装机容量200万千瓦以上。加快发展现代服务业。把服务业发展摆到更加突出的战略位置，坚持市场化、社会化和产业化方向，加强规划引导，创新体制机制，加大政策扶持，强化重点城区、重点园区和重点企业“三大载体”建设，力争服务业占国民经济的比重每年提高一个百分点以上。突出发展物流、信息、金融、商务等生产性服务业，以先进制造业拓展现代服务业，以现代服务业提升先进制造业。培育壮大一批现代物流园区和企业集团。加快发展科技和信息服务业，积极推进电子政务、电子商务和公共信息服务，把“一卡通”作为民心工程加快实施。大力支持金融、保险业发展，拓展商务服务发展空间。推动传统商贸流通转型升级，大力发展新型流通业态，培育发展一批连锁经营龙头企业和大型商贸流通企业集团。培育提升文化旅游、房地产、社区服务等消费性服务业。加大优势文化旅游资源的整合和开发力度，突出特色，建设旅游精品工程，提升淄博旅游的整体形象和吸引力。

　　三、着力提高自主创新能力。把提高自主创新能力作为经济社会发展战略的核心，深入实施科教兴市和人才强市战略，加快建设创新型城市。加强自主创新体系建设。加快建立以企业为主体、市场为导向、产学研相结合的技术创新体系，引导和促进创新要素向企业聚集，加速科技成果向现实生产力转化。坚持走以应用开发为特色的自主创新之路，以引进消化吸收再创新为主渠道，带动原始创新和集成创新。坚持“有所为、有所不为”，在重点领域实现突破。发挥比较优势，在新材料、精细化工、生物医药以及节能减排、信息技术应用开发等领域抓好一批重大科技专项和关键技术攻关，集中力量在应用领域实现重点突破。全面落实推进自主创新的各项政策措施，鼓励大中型企业组建企业技术中心、工程技术研究中心、重点实验室等研发机构，鼓励中小企业建立技术依托，不断提高核心技术开发能力和技术优势。大力营造科技创新良好环境。深化科技管理体制改革，优化科技资源配置。进一步完善政策，加大科技投入，发展创业风险投资，逐步建立多元化的科技投入机制，力争全社会研发经费投入占GDP的比重达到2%以上。加快科技创新服务平台建设，大力培育孵化载体，增强高新区等各类园区的创新服务功能。广泛普及科学知识，加强知识产权的创造、应用和保护。完善和落实发现、培养、引进人才和充分发挥人才作用的政策措施，努力吸引更多优秀人才来淄博创业发展。进一步加强国际科技交流与合作，办好国际科技成果招商洽谈会和新材料技术论坛。力争五年内科技进步对经济增长的贡献率达到60%以上。

　　四、努力建设生态文明。按照党的十七大提出的建设生态文明的新要求，认真贯彻节约资源和保护环境的基本国策，扎实推进资源节约型、环境友好型社会建设。加快形成促进节能减排的体制机制。认真落实节能减排责任制，实行“一票否决”，努力从体制机制上确保节能减排目标的实现。进一步完善能评、环评和“三同时”制度，从源头上控制高耗能、高污染行业发展。分类制定和完善重点产品能耗水耗限额、大型耗能设备能耗限额等指标和标准体系。认真落实差别电价、超标准耗能加价、超计划用水累进加价等政策，积极探索排污总量有偿使用和流转的管理办法，更多地运用市场化手段引导企业节能减排。建立多元化投入机制，加快节能减排新技术、新工艺、新设备的研发、推广和应用，重点组织实施十大节能工程、三个“节能30项”工程和一批环保基础设施建设项目。实施新一轮碧水蓝天行动计划。以建设生态市为目标，以治理结构性污染为重点，深入开展重点流域和重点区域的污染整治。加大环保基础设施投入，加强城镇污水处理厂及配套管网建设。经过五年努力，使城市污水处理100%达到一级排放标准，城市污水处理厂中水100%实现资源化利用，区域内被污染的主要水体100%恢复生态功能。全面完成电厂脱硫改造任务，搞好烟气、粉尘、扬尘治理，不断改善大气质量。全面完成中心城区和区县城市垃圾无害化处理设施建设，城市生活垃圾无害化处理率达到100%。进一步加大执法监管力度，对各类环境违法行为依法“顶格处罚”，决不手软！大力发展循环经济。全面推行清洁生产，加强资源节约利用，培育壮大一批循环经济园区和企业。用好被列为全国首批循环农业示范市的机遇，推进农业循环经济发展。努力形成低消耗、低排放和高效率的节约型发展方式，基本建立起比较完善的循环经济发展体系。加强生态保护和修复。严格保护耕地，加强土地管理，提高土地集约利用水平。大力开展植树造林、荒山绿化，加快四宝山等重点区域生态恢复，实施孝妇河、淄河干流及沂河、猪龙河、涝淄河综合治理工程。理顺机制，加快鲁山、原山、萌山库区、太河库区生态及旅游区规划建设，规划建设马踏湖湿地公园。积极推进生态镇、生态村建设。力争到2012年全市森林覆盖率达到37%。

　　五、打造生态和谐宜居城市。全面贯彻“四个原则”，强化“八个意识”，着力打造特色突出、功能完善、适宜人居的现代化区域中心城市，提高城市现代化水平。充分发挥规划引领控制作用。认真执行新的城市总体规划，加强重点区域专项控制性和修建性详细规划，优化城市功能分区，促进产业发展、城市建设、生态建设相和谐。维护规划的严肃性、权威性，真正把规划落到实处。强化城市设计理念，高水平编制城市风貌规划，加强重点部位的设计控制，更好地体现人文内涵和城市个性。做大做强做美做畅中心城区，协调推进组团城区发展。加快推进中心城区布局调整，力争用三年左右的时间，基本完成东部化工区产业布局调整和南部建材区环境综合整治，推进市高新区、桓台开发区与城市建设的协调发展。积极创造条件，抓好市体育中心、新客运中心等重点项目，推进新城区建设。加快老城区改造步伐，五年内，基本完成旧危房改造和居民小区综合治理。实施重点部位建筑立面整治。完善中心城区路网、管网建设，重点打通断头路，实现主次干道和支路循环、畅通。坚持公交优先，完善公交设施，为广大人民群众提供经济、便捷、舒适的出行服务。加强城市基础设施建设。完善“六纵九横”路网框架，组织实施路网改造升级工程，论证发展城市区际轨道交通。实施清水润城工程，用两年时间完成流经中心城区各条河流的水质净化和河道综合整治。配套完善引黄供水工程，建设“引太入张”城市供水工程。进一步加强供电、供热、供气、供水等市政公用设施建设，不断提高城市服务功能。着力建设便民绿地、环城绿地和城区间绿色长廊，切实提高城市绿化水平，积极争创国家生态园林城市。提高城市管理水平。进一步理顺城市管理体制，强化城市综合执法，推进管理重心下移，建立城市管理长效机制。探索和完善社区管理新模式。深入开展城乡环境综合整治。加快数字城市建设，提高城市管理的信息化、精细化水平。

　　六、深入推进改革开放。加快重要领域和关键环节改革步伐，全面提高开放水平，为经济社会发展提供强大动力。进一步深化各项改革。深化财税、金融、投资、价格等体制改革，积极发展产权、资本、技术、信息、人才等生产要素市场，完善统一开放、竞争有序的现代市场体系。毫不动摇地鼓励、支持非公有制经济发展，加快发展混合所有制经济。继续深化企业改革，完善现代企业制度，增强企业发展活力和竞争力。建立国有资本经营预算制度，加强国有资产监管，提高经营水平。积极发展实体资产证券化，加大政策支持力度，推进企业上市，多渠道扩大直接融资比重，五年内全市上市公司数量达到30家，资本市场融资规模新增150亿元以上。推进事业单位分类改革，完善事业单位管理体制。理顺市与区县经济管理权限，促进区县财权与事权相统一。充分利用国家促进资源型城市可持续发展的政策，加速解决我市老工矿区改革发展所面临的矛盾和问题。大力发展区县域经济，加强对区县域经济发展的规划引导，注重发挥比较优势，进一步形成特色鲜明、优势互补、协调联动、错位发展的格局。继续做好突破高青、支持沂源加快发展工作。全面提高利用外资水平。坚持市外就是外，充分发挥各类存量资源优势，抢抓机遇，精心策划一批大项目、好项目，提高专业招商水平。积极吸引外资重点投向高新技术产业、装备制造业、现代服务业、节能环保产业、现代农业和基础设施等领域，推动节能减排、自主创新和产业升级。着力引进先进技术和管理，吸引跨国公司到我市建立研发中心、采购中心，推动骨干企业集团与国内外大企业集团开展战略合作。创新利用外资方式，鼓励企业境外上市和股权融资。加快转变外贸增长方式。把调整优化出口商品结构与节能减排结合起来，扶持机电、高新技术产品和自主品牌、自主知识产权产品扩大出口，推进加工贸易转型升级。利用国家大力支持进口的政策，积极扩大先进技术装备和重要原材料进口。加快发展服务贸易，积极承接国际服务外包。进一步扩大对外经济技术合作。鼓励我市具有比较优势的产业“走出去”，支持企业在研发、生产、销售等方面开展国际化经营。大力发展劳务输出和境外工程承包。积极参与山东半岛城市群、省会城市群经济圈以及环渤海经济圈等区域经济合作。继续办好陶博会。做好对口支援工作。

共建共享和谐淄博

　　扎实推进以改善民生为重点的社会建设，发展社会事业，扩大公共服务，完善社会管理，促进公平正义，使全市人民学有所教、劳有所得、病有所医、老有所养、住有所居。

　　一、千方百计扩大就业增加居民收入。全面落实积极的就业政策，加快建立健全面向全体劳动者的职业教育培训制度和面向所有困难群众的就业援助制度，力争每年新增城镇就业再就业9万人以上。完善支持自主创业、自谋职业的政策措施，着力促进以创业带动就业。稳步增加公益性就业岗位，帮助零就业家庭和困难人员实现就业。加快建设统一规范的人力资源市场，做好高校毕业生就业工作，促进城乡劳动者平等就业。继续组织实施农村劳动力培训“阳光工程”，切实维护劳动者特别是农民工的合法权益，构建和谐劳动关系。健全劳动、资本、技术、管理等生产要素按贡献参与分配的制度，逐步提高居民收入在国民收入分配中的比重、劳动报酬在初次分配中的比重，创造条件让更多群众拥有财产性收入。完善企业职工工资增长指导线和最低工资保障制度，全面实行劳动合同和工资集体协商制度，建立企业职工工资正常增长机制和支付保障机制，稳步提高低收入者收入，扩大中等收入者比重。有效调节过高收入，取缔非法收入，规范分配秩序。

　　二、建立健全覆盖城乡的社会保障体系。加快构筑以社会保险、社会救助、社会福利为基础，以基本养老、基本医疗、最低生活保障制度为重点，以慈善事业、商业保险为补充的社会保障体系。着力推进以混合所有制、非公有制经济组织的从业人员、灵活就业人员和农民工为重点的社会保险征缴扩面。建立新型农村养老保险制度，完善不稳定就业农民工和被征地农民养老保险。加快实现城镇居民基本医疗保险的全覆盖，巩固、完善新型农村合作医疗制度，逐步提高筹资水平，扩大保障范围。加强社会保险基金监管，确保基金安全。健全城乡居民最低生活保障制度和社会救助体系，完善“五保户”集中供养等制度。大力发展社会福利、慈善、残疾人、老龄、红十字事业。加强住房公积金管理，强化房地产市场监管。健全廉租住房制度，加快经济适用房和廉租房建设，努力解决中低收入群体的住房困难。

　　三、加快发展社会事业。把教育摆在优先发展的战略地位，办好公平质优、群众满意的教育。完善义务教育经费保障机制，逐步免除义务教育阶段学生课本费，健全学生资助制度，保障经济困难家庭、进城务工人员子女平等接受义务教育。调整优化城乡中小学布局，解决城区部分学校班额过大问题，促进义务教育均衡发展。加强教师队伍建设，大力发展素质教育，加快教育现代化建设。进一步提高高中段教育普及率，大力发展职业教育，提高高等教育质量。重视学前教育，关心特殊教育。鼓励和规范社会力量办学。加强青少年宫等青少年校外教育培训活动基地建设。发展远程教育和继续教育，建设学习型城市。坚持公共医疗卫生的公益性质，以城乡居民人人享有基本医疗卫生服务为目标，深化医疗卫生体制改革，加强农村卫生和城市社区卫生服务网络建设，力争用三年左右时间，完成农村卫生室改造和城市社区卫生服务机构新建及改扩建，为人民群众提供安全、有效、方便、价廉的医疗卫生服务。健全重大疾病防控体系，提高公共卫生事件应急处置能力。扶持中医药事业发展。积极发展妇幼卫生事业。稳定低生育水平，提高出生人口素质，改善人口结构。广泛开展爱国卫生运动，巩固国家卫生城市创建成果。做好第11届全运会协办和第22届省运会承办工作，促进全民健身运动开展和竞技体育水平提高。加强民兵预备役和国防建设，深入开展双拥共建活动。建设殷实小康、构建和谐淄博，必须进一步强化审计、统计、民族、宗教、外事、侨务、对台、气象、人防、档案、仲裁、史志、防震减灾等工作。

　　四、促进文化繁荣发展。坚持先进文化前进方向，大力推动社会主义核心价值体系建设，弘扬爱国主义、集体主义、社会主义思想，着力塑造“诚信、务实、开放、创新”的城市精神。建设和谐文化，培育文明风尚。以创建全国文明城市为目标，广泛开展群众性精神文明创建活动，加强社会公德、职业道德、家庭美德、个人品德建设，坚持正确导向，强化网络文化建设和管理，营造良好网络环境，形成知荣辱、讲正气、促和谐的良好社会风尚。深入推进“诚信淄博”建设，以政务信用为关键、商务信用为重点、社会信用为基础，建立和完善社会信用体系。重视城乡、区域文化协调发展，着力丰富农村、偏远地区、进城务工人员的精神文化生活。深化文化体制改革，增加公益性文化事业投入，建设市图书馆新馆等设施，全面完成区县文化中心及乡镇综合文化站建设，推进文化信息资源共享工程，形成较为完善的公共文化服务体系，保障人民群众的基本文化权益。繁荣发展新闻出版、广播影视、文学艺术事业。加强文物和非物质文化遗产保护与利用，延续历史文脉，提升淄博文化的影响力。大力发展文化产业。抓住省建设齐文化产业园的机遇，深度开发以齐文化为代表的优秀传统文化资源。加快培育文化产品市场和要素市场，加强重大文化项目和文化品牌建设，扶持会展、文博、创意、动漫等新兴文化产业，培育一批主业突出、实力雄厚、具有较强竞争力的文化企业集团，提升文化产业规模和质量。

　　五、全面加强和改进社会管理。坚持党委领导，强化政府责任，加大社会协同和公众参与力度，提高社会管理水平。妥善处理人民内部矛盾，坚决纠正损害群众利益的行为，最大限度激发社会创造活力，最大限度增加和谐因素，最大限度减少不和谐因素。完善信访制度，畅通群众诉求渠道，综合运用调解、仲裁、行政复议等方式，有效化解各类矛盾。加强农村基层组织和城市社区建设，健全基层管理体制，推进重心下移。搞好流动人口管理和服务。深入推进平安淄博建设，加强社会治安综合治理，健全完善社会治安防控体系，改革和强化城乡社区警务工作，依法防范和严厉打击违法犯罪活动。建立健全应急管理体制，有效预防和处置各类突发事件，维护社会稳定。树立安全发展理念，加强安全生产，防止重特大事故发生。健全完善食品、药品质量安全监管长效机制，保障群众饮食、用药安全。

深入推进服务型政府建设

　　面对改革开放和现代化建设的新形势，面对殷实小康、和谐淄博建设的新要求，面对全市人民对美好生活的新期待，各级政府要以更加开阔的视野、更加饱满的热情、更加扎实的工作，深入推进为民、务实、清廉的服务型政府建设，努力开创政府工作的新局面。

　　一、加快政府职能转变。深化行政管理体制改革，加快建立权责一致、分工合理、决策科学、执行顺畅、监督有力的行政管理体制。搞好经济调节和市场监管，维护公平竞争的市场秩序，保障经济健康运行。着力加强社会管理和公共服务，将更多公共资源集中到改善人民生活、促进社会和谐的行业和领域。切实推进政企分开、政资分开、政事分开、政府与市场中介组织分开，规范行政行为。进一步理顺各级政府及其部门间的事权关系，减少行政层次，降低行政成本，努力消除职能交叉、多头管理、政出多门。深化行政审批制度改革，进一步精简审批事项，简化审批程序，加强各级行政服务中心和乡镇便民服务中心建设，推动行政便民服务向基层延伸。

　　二、推进政府管理创新。健全完善政府决策机制，落实市民建议征集、重大事项公示、专家咨询、社会听证、决策评估和社情民意调查等制度，使决策更加符合淄博实际、符合群众根本利益。健全工作考核机制，探索推行符合科学发展观要求、有利于促进发展方式转变、体现发展实绩的绩效考核措施。加快电子政务建设，办好政府门户网站，扩大网上便民公共服务范围，推进信息资源共享。大力推进政务公开，重点推进公益性强、公众关注程度高的政府部门信息公开，切实保障群众的知情权、参与权、表达权和监督权。继续推行首问负责、服务承诺、限时办结等制度。大力精简会议和文件，提高管理效能。强化制度信用、政策信用和程序信用，做到政策透明、制度公开、程序规范，全面提升政府诚信。

　　三、提高依法行政水平。加强和改进政府立法工作，不断完善地方性法规和行政规章体系。深化行政执法体制改革，扩大综合执法范围，在更多领域实行相对集中行政处罚权，进一步规范行政处罚自由裁量权。全面落实行政执法责任制，健全执法评议考核、行政赔偿和执法过错责任追究制度，保障严格执法、文明执法、公正执法。大力加强行政执法监督，坚决纠正部门和行业不正之风，以严格的管理、严明的纪律，保证政府的执行力和公信力。深入开展法制宣传教育，弘扬法治精神，形成自觉学法守法用法的社会氛围。大力加强基层民主政治建设，完善厂务公开、村务公开等制度；推进城市居民自治，扩大社区居委会直选范围。各级政府要自觉接受同级人民代表大会及其常务委员会的工作监督和法律监督，积极支持人民政协履行政治协商、民主监督、参政议政职能，支持工会、共青团、妇联等群众团体开展工作，认真办理人大代表议案、建议和政协委员提案，努力推动政府民主法治进程。

　　四、建设高素质公务员队伍。全体政府公务员要按照“认真、专业、务实”的政风要求，加强学习、勇于实践，做工作中的行家里手，不断提高解决复杂问题、推进工作落实的能力。要强化宗旨意识和立党为公、执政为民理念，把更多的时间和精力用在调查研究上，用在帮助基层和群众解决实际问题上，全心全意为群众办实事、谋福祉。进一步培育科学精神，坚持以科学的态度研究工作，以科学的精神推进决策，以科学的办法解决问题，使政府工作经得起实践、群众、历史的检验。加强反腐倡廉建设，落实廉政建设责任制，建立健全教育、制度、监督并重的惩治和预防腐败体系，严肃查处违法违纪案件。全体政府工作人员特别是领导干部，要强化忧患意识、公仆意识、节俭意识，培养健康的生活情趣，讲操守、重品行，始终保持谦虚谨慎、艰苦奋斗的作风，努力建设一支政治坚定、业务精通、清正廉洁、作风优良的公务员队伍。为政之道，贵在实干，重在落实。全体政府工作人员要把求真务实抓落实作为工作准则来遵循，作为日常习惯来培养，作为一种能力来提高，不搞花架子，不做表面文章，不弄虚作假，真正做到想抓、会抓、抓好，努力开创各项工作新局面。

　　各位代表！2008年是全面贯彻落实党的十七大作出的战略部署的第一年，也是新一届政府工作的开局之年，做好今年的工作意义重大。今年全市经济社会发展的主要预期目标建议为：国内生产总值增长12%，地方财政收入增长16%，规模以上固定资产投资增长20%;主要约束性目标建议为：万元生产总值综合能耗降低5%，化学需氧量和二氧化硫排放量分别削减4.5%和8%。具体工作安排和建议，将在市政府向大会提交的计划报告和财政报告中阐述。概括而言，工作中要以党的十七大精神为指导，以科学发展观统领全局，全面贯彻落实中央经济工作会议和省委九届三次全会精神，按照市十次党代会和市委十届三次全体会议的部署，坚持稳中求进、主动有为、好字优先、又好又快，积极推进工作指导转变和发展方式转变，更加注重发展的质量和效益，更加注重节能环保和自主创新，更加注重社会建设和民生改善，努力在重点领域和关键环节实现新的突破，为完成本届政府五年任期目标开好局、起好步。一是着力优化经济结构，实现又好又快发展新突破;二是着力推进节能降耗和环境保护，实现生态文明建设新突破;三是着力统筹城乡发展，实现城市建设和新农村建设新突破;四是着力推进体制机制创新，实现改革开放新突破;五是着力推进以改善民生为重点的社会建设，实现共建共享和谐淄博新突破;六是着力推动文化大发展大繁荣，实现先进文化建设新突破;七是着力加强政府自身建设，实现服务型政府建设新突破。同时，扎扎实实为人民群众办好十个方面的实事：(1)实施廉租住房货币补贴制度，全面解决城市低收入家庭的住房困难;(2)建立和完善城镇居民基本医疗保险制度，确保城镇居民人人享有基本医疗保险保障;(3)建立健全家庭经济困难学生资助政策体系，促进教育公平;(4)实施食品药品质量安全工程，确保城乡居民饮食用药安全;(5)实现城市社区卫生服务全覆盖;(6)完成农村中小学危房改造工作，启动农村中小学仪器设备更新工程，促进农村基础教育事业健康和谐发展;(7)实现城市生活垃圾无害化处理和全市秸秆无焚烧;(8)大力发展城市公共交通，对中心城区居民持IC卡乘车实行低价优惠;(9)全面推进社会福利事业发展，建设淄博市老年福利服务中心和淄博市儿童福利院，完成农村残疾人“安居工程”;(10)健全完善社会治安防控体系，实施治安安民工程。

　　各位代表！带着全市人民赋予的重任和期盼，我们满怀信心踏上了建设殷实小康、构建和谐淄博的新征程。让我们更加紧密地团结在以胡锦涛同志为总书记的党中央周围，高举中国特色社会主义伟大旗帜，在中共淄博市委的坚强领导下，解放思想，开拓奋进，为夺取全面建设小康社会新胜利而努力奋斗！
